# The NorthMom study: population-based follow-up of women in a birth cohort to assess pregnancy exposures and effects on breast cancer risk factors

**DOI:** 10.1186/s12889-026-29622-0

**Published:** 2026-09-24

**Authors:** Alice Fredriksson, Richard Lundberg-Ulfsdotter, Marie-Therese Vinnars, Anna Oudin, Bethany Van Guelpen, Christina E. West, Magnus Domellöf, Wendy Yi-Ying Wu, Karin Dembrower, Sophia Harlid

**Affiliations:** 1 Department of Diagnostics and Intervention, Oncology, Umeå University, Umeå, 90187 Sweden; 2 Department of Clinical Sciences, Pediatrics, Umeå University, Umeå, Sweden; 3 Department of Clinical Sciences, Obstetrics and Gynecology, Umeå University, Umeå, Sweden; 4 Department of Epidemiology and Global Health, Umeå University, Umeå, Sweden; 5 Division of Occupational and Environmental Medicine, Department of Laboratory Medicine, Lund University, Lund, Sweden; 6 Wallenberg Centre for Molecular Medicine, Umeå University, Umeå, Sweden; 7 Breast Imaging Unit, Department of Radiology, Capio Sankt Göran Hospital, Sankt Göransplan, Stockholm, Sweden; 8 Department of Medical Epidemiology and Biostatistics, Karolinska Institutet, Stockholm, 171 77 Sweden

**Keywords:** NorthMom, NorthPop, Environmental exposures, Women’s health, Pregnancy, Breast cancer risk, Mammographic breast density, Epidemiology, Risk factors

## Abstract

**Background:**

The NorthMom Study (NorthMom) is an observational, population-based cohort designed to determine how the environment during pregnancy affects later health risks among women. The specific focus of NorthMom is breast cancer risk, evaluated through intermediate markers such as breast density, and additional outcomes may be studied in the future.

**Methods:**

NorthMom is nested in the larger NorthPop Birth Cohort Study (NorthPop), which was initiated in Västerbotten County, Sweden in 2016 and closed recruitment in 2025. All women previously enrolled in NorthPop are invited to join NorthMom after turning 40 years of age. Women who have moved from the county, have withdrawn or declined follow-up from NorthPop, or are pregnant at the time of invitation are considered ineligible. Those who agree to participate visit the Clinical Research Center at the University Hospital of Umeå in Västerbotten, Sweden. A study nurse collects a blood sample and additional health data, including body composition by Dual-Energy X-ray absorptiometry (DXA). To assess breast density, mammographic images from the Swedish national breast cancer screening program are acquired and evaluated both manually (by a consultant radiologist) and automatically using the Laboratory for Individualized Breast Radiodensity Assessment (LIBRA) software. Measures include classification according to BI-RADS 4 (1–4) and BI-RADS 5 (A-D), as well as continuous densities (%) and dense area (cm^2^). Health-related and environmental exposure data are collected by NorthPop during pregnancy and requested for secondary use in NorthMom. Available exposures include geo-spatial data on ambient air pollution, traffic noise, and green environments. For subsets of women, analyses of chemicals in plasma samples collected during pregnancy are available. Additional chemical exposomics analyses and blood biomarker analyses, including proteomics and metabolomics, may be relevant in the future.

**Discussion:**

Analysis of data collected as part of NorthMom and NorthPop will be used to assess relationships between environmental exposures during pregnancy and women’s later health, with a focus on breast cancer risk factors such as breast density after age 40.

**Trial registration:**

Registered in Researchweb, 19 August 2026 (project number 287619).

**Supplementary Information:**

The online version contains supplementary material available at https://doi.org/10.1186/s12889-026-29622-0.

## Background

Breast cancer incidence is increasing among women worldwide, being true for both premenopausal and postmenopausal disease [1]. Global changes to reproductive factors, such as increasing age at first pregnancy and lower parity, may contribute; however, they cannot fully explain the rise in cases [2, 3]. Previous studies focusing on environmental drivers of disease, including exposure to air pollution, traffic noise, and endocrine-disrupting chemicals (EDCs), often lack clearly characterized populations with detailed clinical information and high-quality exposure assessments collected during relevant time periods, hampering their ability to detect associations [4, 5]. To better understand the connections between our environment and breast cancer development, we are therefore planning studies in which environmental exposures occurring during important time periods, such as pregnancy, are evaluated in a well-defined cohort in Northern Sweden.

Environmental exposures may be particularly potent during so-called “windows of susceptibility” (WoS), including prenatal development, puberty, pregnancy, and menopause [6]. WoS for breast cancer are marked by substantial changes in mammary gland structure, temporarily increasing vulnerability to environmental factors that can influence later breast cancer risk [6]. Pregnancy is an understudied WoS during which breast tissue undergoes several critical stages of development and maturation, driven by hormonal fluctuations [7]. This makes the gland sensitive to exposures, for example, through contact with chemicals that interact with hormone receptors [8, 9]. Such exposures could trigger biological effects, including alterations in breast density [10, 11], reduced lactation length [12, 13], and possibly long-term changes in endogenous biomarkers and gene expression [14], all of which may serve as potential mediators between exposure and breast cancer development.

High breast density (defined as the proportion of fibroglandular tissue within the breast) is an independent risk factor for breast cancer, and it also reduces the sensitivity of mammographic screening by masking underlying lesions [15]. A woman’s breast density is influenced by hormonal, genetic, and environmental factors, and it may serve as an intermediate marker linking environmental exposures to later disease development. However, few studies have investigated associations between breast density and environmental exposures, particularly if occurring during WoS [10].

Breastfeeding duration represents another potential pathway linking pregnancy exposures to later breast cancer risk. Breastfeeding has consistently been associated with reduced breast cancer risk, and it is widely recognized as a protective factor in cancer prevention recommendations [16]. Lactation ability depends on coordinated hormonal and developmental processes that occur during pregnancy, especially during the later stages of gestation [17]. Previous studies have demonstrated associations between Per- and polyfluoroalkyl substances (PFAS) exposure and shorter breastfeeding duration [12, 13], although findings have not always been consistent [18].

In addition, environmental exposures may induce changes in the internal exposome, including persistent alterations in the proteome, metabolome, and epigenome [19]. Epigenetic alterations, such as differential DNA methylation, could, for example, influence long-term gene expression patterns and future susceptibility to disease [20]. Some evidence from animal and epidemiological studies suggests that prenatal exposure to elevated estrogen levels may lead to persistent epigenetic changes later in life [21, 22]. However, few studies have investigated how environmental exposures during pregnancy may be linked to long-term changes to the internal exposome and potential implications for breast cancer risk.

Although pregnancy may serve as a highly influential time period when it comes to breast cancer susceptibility, important knowledge gaps remain regarding how environmental exposures during pregnancy impact breast cancer-related markers and later disease risk. Longitudinal cohorts with detailed exposure assessment, biological sampling, and long-term follow-up are therefore needed to improve understanding of these relationships across the life course.

To address this, we have established The NorthMom Study (NorthMom), which focuses on environmental exposures during pregnancy and their effects on breast cancer-related outcomes and mechanisms. By integrating detailed exposure assessment, biological sampling, reproductive data, and imaging-based outcomes, the study aims to improve understanding of the environmental determinants of breast cancer and to inform future prevention and risk stratification strategies.

## Methods/design

### Design, setting, and study aim

NorthMom is a new sample and data collection nested within the population-based NorthPop Birth Cohort Study (NorthPop) in Västerbotten County, Sweden [23]. Between 2016 and 2025, all pregnant women in Västerbotten County, Sweden, were invited to participate in NorthPop, together with their partners. Recruitment occurred at the time of a prenatal ultrasound examination, most commonly the routine ultrasound examination, between gestational weeks 14–24 and has been previously described [23]. During pregnancy, NorthPop collected extensive longitudinal data from questionnaires, biological samples, and environmental assessments. Approximately 10 000 pregnant women and their partners were recruited to NorthPop and will now be followed, along with the child of the index pregnancy, at least until the child turns seven [23]. NorthMom serves as a separate follow-up of the pregnant women recruited to NorthPop, and while the main aim of NorthPop is to investigate exposures during gestation and early life and how they influence children’s development, NorthMom examines how exposures during pregnancy affect women’s later health.

### Recruitment and primary data collection

**Fig. 1:**
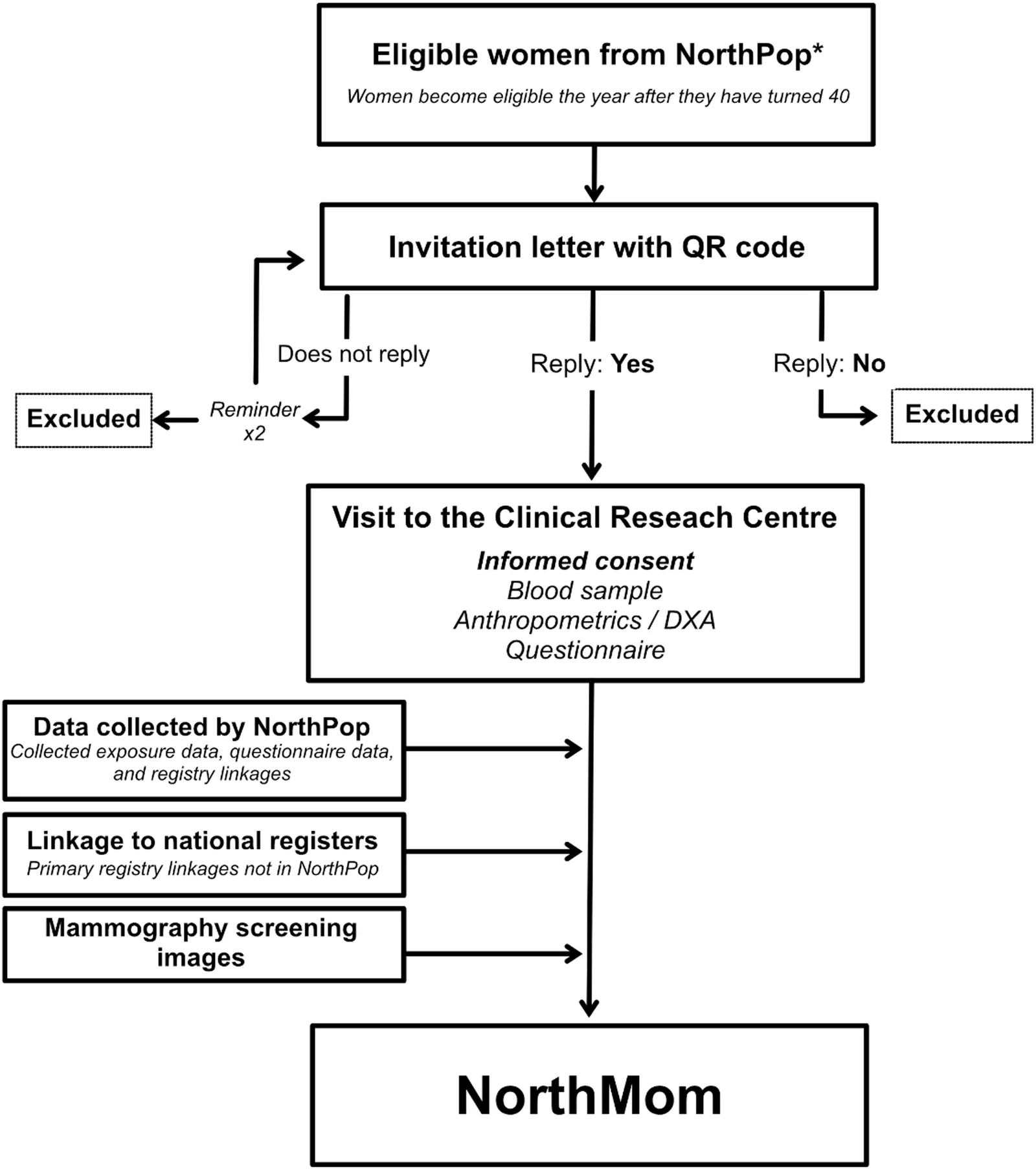
Flowchart showing recruitment and data acquisition to the NorthMom Cohort. *Excludes women who moved from the county, dropped out of NorthPop, declined further follow-up, or are pregnant at the time of invitation

All women in NorthPop are invited to participate in NorthMom the year after they have turned 40. The list of eligible participants is updated annually to include additional women as they reach the required age. Women who have moved out of Västerbotten County, withdrawn from NorthPop, declined further follow-up, or are currently pregnant are considered ineligible. All eligible women receive a letter inviting them to participate in NorthMom; if they do not answer, two reminders (approximately six weeks apart) are sent, after which they are excluded from the study (Fig. 1). If they wish to participate, they can register using a QR code provided in the letter (a translation of the invitation is available in the supplementary materials). Following registration, a health care professional at the Clinical Research Center at the University Hospital of Umeå makes contact to schedule an in-person visit. At the visit, all women provide written informed consent and are thereafter included in the study. A venous blood sample is subsequently collected in EDTA tubes and aliquoted into plasma, buffy coat, and erythrocyte fractions, which are stored at -80 °C at the regional biobank (Biobanken norr). A study nurse collects anthropometric measures such as height and weight. The participants also undergo a Dual-Energy X-ray absorptiometry (DXA) scan, which measures body composition, including body fat percentage and fat distribution. Finally, women fill out a short questionnaire focusing on reproductive history and previous or current hormone therapy or contraceptive use (Table S1). All collected data are saved and managed using REDCap electronic data capture tools hosted at Umeå University [24, 25] (Table 1). A flowchart of the full recruitment process is depicted in Fig. 1.

**Table 1:** Examples of data collected at the NorthMom study visit

Data collected	Description	Collection method
Blood sample	Venous sample collected in EDTA tubes	Collected by study nurse
Anthropometrics	Height, weight, and BMI	Collected by study nurse
Fat percentage	Total fat percentage	From DXA scan
Android/Gynoid fat ratio	The abdominal by hip and thigh fat ratio	From DXA scan
Visceral fat area	Amount of belly fat	From DXA scan
Age at menarche and menopause*	Age at first and last menstrual periods	From questionnaire
Menstrual cycle information	Cycle length and regularity	From questionnaire
Parity	Total number of pregnancies	From questionnaire
Time to pregnancy	Time from trying to successful pregnancy	From questionnaire
Fertility treatments	Medical help for fertility issues	From questionnaire
Hormone use	Previous and current hormone use	From questionnaire

*Only relevant for women who are postmenopausal at the visit

### Secondary data retrievals

As all NorthMom women previously participated in NorthPop [23], extensive information about environmental exposures during pregnancy has already been collected prospectively. This data contains information on lifestyle during pregnancy (such as detailed dietary and physical activity data), medications, mental health, and home and work environments, as previously described [23]. Measures or modeled environmental exposures during pregnancy are also available for all or subsets of women; these include, but are not limited to, air pollution and traffic noise exposure, residential access to green spaces, and plasma levels of persistent organic pollutants (POPs). NorthPop also links to multiple national and quality registers, including the national patient register [26] and the pregnancy register [27], enabling NorthMom to use this data. Finally, blood and urine samples collected during pregnancy, as part of NorthPop, can also be requested for use in NorthMom studies. All sample and data retrievals from NorthPop are conducted on a study-by-study basis and require separate approvals from both the Swedish Ethical Review Authority and the NorthPop Steering Committee.

NorthMom may also perform linkages to national registers that are separate from those conducted by NorthPop. These include additional linkages to the national prescribed drug register [28], the national cause of death register [29], the Swedish national breast cancer register (NKBC) [30], the national cancer register [31], and the Swedish multigenerational register [32] (in order to obtain data on family history of cancer). Planned and ongoing registry linkages are listed in Table 2.

**Table 2:** Register linkages, planned and ongoing

Register name	Description
**NorthPop Linkages, secondary access**	
Maternity care center records	Assessments during pregnancy
The Longitudinal Integrated Database for Health Insurance and Labor Market Studies (LISA)	Socioeconomics, including income and education
The National Prescribed Drug Register	Prescribed drugs during the NorthPop study period*
The Swedish Pregnancy Register	Pregnancy, childbirth, and neonatal outcomes
The National Patient Register	Data on health care episodes during the study period
The Swedish Geographical Database	Geographic data, including residential addresses
**NorthMom Linkages, primary access**	
The Multigenerational Register	Identification of first-degree relatives
The Swedish Cancer Register	Any cancer diagnosis
The Swedish Breast Cancer Register (NKBC)	Detailed data on breast cancer diagnoses
The Medical Birth Register	Information on previous and total parity
The National Quality Registry for Assisted Reproduction (Q-IVF)	Use of IVF or assisted reproduction
The National Prescribed Drug Register	Prescribed drugs outside of the NorthPop study period*

*NorthPop obtains prescribed drugs data from the time of pregnancy until the child is 7 years old

From the Västerbotten health region, NorthMom retrieves radiological images taken as part of the national population-based mammography screening program (further described in the supplementary materials). Screening images are used to classify breast density both automatically [33, 34] and via subjective assessment (Table 3). The subjective assessments are performed by a consultant breast radiologist Karin Dembrower (KD), who retrospectively records the date of the breast examination, reports subjective breast density, and classifies breast density according to the Breast Imaging Reporting and Data System (BI-RADS) (both versions 4 and 5 for all images) [35]. The BI-RADS^®^ 4th edition categorizes mammographic breast density into four specific groups based on the approximate percentage of fibroglandular tissue visible on the mammogram. Category 1: Almost entirely fatty < 25% glandular tissue. Category 2: Scattered fibroglandular densities, 25% − 50% glandular tissue. Category 3: Heterogeneously dense, 51% − 75% glandular tissue. Category 4: Extremely dense tissue > 75% glandular tissue. The BI-RADS^®^ 5th edition classifies breast density (composition) into four categories. In this edition, percentage thresholds from previous editions were removed. Instead, the categories are visually assessed based on the amount of glandular/fibrous tissue and the likelihood that it could obscure a small tumor (the masking effect). Category (A) Almost entirely fatty: Breasts are composed mostly of fat with very little dense tissue; masses are highly visible. Category (B) Scattered areas of fibroglandular density: There are scattered patches of dense tissue, but most of the breast is fatty. Category (C) Heterogeneously dense: The breast has widespread areas of dense tissue, which may lower mammogram sensitivity and obscure small masses. Category (D) Extremely dense: The tissue is almost entirely dense, which significantly lowers the sensitivity of mammography [36].

**Table 3:** Data extracted from mammographic images

Variable	Description
**Subjective measures**	
Examination date	Date of mammographic examination
BI-RADS 4 classification	Density classified according to BI-RADS 4 (1–4)
BI-RADS 5 classification	Density classified according to BI-RADS 5 (A-D)
Continuous density	Classified density between 1-100
Focal density	Any sign of focal density
Automatic measures*	
Percent density, total	Percent density based on all images
Percent density MLO only	Percent density based on MLO images only
Dense area, total	Dense area (cm^2^), based on all images
Dense area, MLO only	Dense area (cm^2^), based on MLO images only

* Evaluated using the Laboratory for Individualized Breast Radiodensity Assessment (LIBRA) software

*MLO* Mediolateral Oblique angle images

Automatic assessments of breast density are performed using the Laboratory for Individualized Breast Radiodensity Assessment (LIBRA) software for automatic density measurements [37]. From LIBRA, information on mean dense area (cm^2^) and percent density is extracted for each woman. To account for possible discrepancies between subjective and automated density measures, we include measures of mean dense area and percent density at the Mediolateral Oblique (MLO) angle, which covers the entire breast (supplementary figure S1). Correlations between BI-RADS assessments, subjective percent density, and automatic density measures are continuously assessed. For women with multiple screening occasions, changes in density measurements over time are also evaluated and documented in NorthMom.

## Planned future analyses

We foresee extensive future analyses based on the data and blood samples currently being collected in NorthMom, as well as through linkage to other cohorts, biobanks, and registries over the life course of the participants. Because blood samples are collected as part of both NorthMom and NorthPop, there is ample opportunity to analyze internal biological markers of environmental exposures. In addition, genetic analyses for mothers and children in NorthPop are planned and will be reusable in NorthMom. Aside from the primary research questions on the environment during pregnancy and breast cancer risk factors, women will be followed (by registry linkage) for exposures and relevant health outcomes throughout and after the menopausal transition. With respect to additional cohort linkage, Västerbotten has a long tradition of population-based sampling and data collection, with one of the leading examples being the Northern Sweden Health and Disease Study (NSHDS) [38]. Over 70% of the population of Västerbotten over 40 years of age are included in NSHDS, mainly through the Västerbotten Intervention Programme (VIP), a community prevention intervention for cardiometabolic disease within primary health care [39]. Residents of Västerbotten attend VIP at ages 40, 50, and 60, at which time they are also invited to provide a research blood sample. Over time, a substantial proportion of NorthMom participants can be expected to enter NSHDS. In the future, it may therefore be possible to obtain detailed information (including blood samples) on exposures across major phases of life that can affect women’s health, covering pregnancy (NorthPop), perimenopause (NorthMom), menopause (NSHDS) and postmenopause (NSHDS).

## Data analyses and power calculations

Analyses in NorthMom will focus on identifying associations between environmental exposures during pregnancy/postpartum and factors known to be associated with breast cancer risk. The primary outcome will be breast density measured at approximately 40 years of age, but other outcomes may be assessed in future studies. Environmental exposures during pregnancy will include both externally assessed exposures, such as air pollution, traffic noise and proximity to green environments, and internally assessed exposures including biomarker-based measures.

As an example, associations between environmental exposures during pregnancy and continuous breast density at age 40 may be assessed using linear regression models. Regression coefficients for standardized continuous exposures will then represent the absolute difference in breast density associated with a 1-SD increase in exposure, with adjustments for prespecified potential confounders.

Breast densities will also be evaluated using the BI-RADS^®^ categories. When using the BI-RADS^®^ 5th categories (A-D), we will first treat them as ordinal outcomes and analyze them with ordinal regression. The categories may subsequently be reclassified into lower- and higher-density groups for analysis as a binary outcome using logistic regression.

In addition to the primary analysis at age 40, repeated breast density measurements obtained at regular screening intervals (e.g., at 40, 42, and subsequent ages) may be used to examine longitudinal patterns in breast density. Mixed-effects models will then be used to account for within-woman correlation across repeated measurements. Age will be used as the longitudinal time scale, with woman-specific random intercepts and, where supported by the data, random slopes. Interactions between environmental exposures and age will be examined to assess whether environmental exposures during pregnancy are associated with differences in the rate of change in breast density over time.

For analyses of associations between the exposures and continuous breast density (the primary outcome in the study), we conducted a sample size calculation based on breast density at age 40 (performed in R using the ss.SLR() function in the powerMediation package). Pilot data showed a standard deviation of 19.71% points for breast density. Assuming a two-sided significance level of 0.05, 1049 women with available exposure and breast density data would provide 80% power to detect a 1.7%-point difference in breast density per 1-SD increase in exposure.

## Data storage and management

All data collected at the NorthMom study visit or extracted from screening mammograms are stored in a REDCap database hosted by the unit for IT support and system development at Umeå University (ITS). Datasets extracted from REDCap for analytical purposes are stored and analyzed on secure servers managed by ITS and backed up weekly. Data collected by NorthPop is also stored on ITS servers, with access and backups controlled by the NorthPop data manager. Once recruitment and data collection for NorthMom are finalized, all data will be transferred to NorthPop and managed within its infrastructure. All data handling in NorthMom is performed in accordance with the General Data Protection Regulation (GDPR).

## Access to data

All future access to NorthMom data will be handled by the NorthPop steering committee and require study-specific ethical approval from the Swedish Ethical Review Authority.

## Discussion

NorthMom is a follow-up study that recruits women previously participating in NorthPop. In NorthMom, women are followed from pregnancy to assess future health outcomes with a specific focus on breast cancer risk. The setting enables the use of already collected data on pregnancy exposures, including both spatial data and biological measurements. Additionally, since women are primarily recruited before the onset of menopause, it will be possible to capture associations with both pre- and, eventually, postmenopausal breast density. Previous studies have indicated that exposure to endocrine disruptors early in life can affect future breast density [35], and NorthMom will be well-suited to address research questions regarding such associations. Planned biomarker analyses will also be instrumental in upcoming studies of the internal exposome and may improve our understanding of how it affects health risks both pre- and post-menopause.

Multiple environmental exposures are already covered in NorthMom, including traffic noise, residential access to green spaces, several chemical measurements, and ambient air pollution. Air pollution is one of the most extensively studied environmental exposures in breast cancer epidemiology, and nitrogen dioxide (NO₂), a commonly used marker of traffic-related air pollution, has been associated with both pre- and postmenopausal breast cancer risk [40–42]. Endocrine-disrupting chemicals (EDCs) are a group of environmental exposures of particular interest in breast cancer epidemiology, as they are capable of interfering with hormonal signaling pathways and have been associated with reproductive disorders, metabolic disease, and hormone-sensitive cancers, including breast cancer [8, 9, 11]. Subsets of women in NorthMom have available measures of, e.g., POPs in serum samples collected during pregnancy. In the future, we plan to measure chemical exposures in all enrolled women using plasma exposomics [43].

During the first year, NorthMom had a recruitment rate of 36.6% with 442 out of 1207 invited women agreeing to participate (74 women (6.1%) declined to participate, and 691 women (57.2%) did not respond to the invitation or subsequent reminders). There are a few discrepancies related to mammography images, as some women had not yet been invited to participate in the screening program at the time of recruitment. This mismatch in overlap may decrease over time as more women attend mammography screening. However, as outlined in the description of the Swedish mammography screening program (supplementary materials), about 20% of women do not attend the program. The percentage of women lacking available mammograms in NorthMom is, however, expected to be smaller than in the average population, as they have previously participated in both NorthPop and NorthMom and may, due to inherent characteristics, be expected to also participate in the screening program [44].

As of the end of 2026, funding for NorthMom will allow recruitment to continue until at least the end of 2028. By then, more than 3 000 women in NorthPop will have turned 40, and we should have approximately 1 000 participants in NorthMom (assuming a continued recruitment rate of at least 35%). If additional funding is obtained, recruitment will be prolonged. However, the youngest women in NorthPop at the moment have not yet turned 30, so recruitment would need to continue for approximately 15–20 more years before all women have had a chance to be invited.

Specific strengths of the NorthMom study include collecting data on body fat distribution and percentage using the DXA scan [45]. Adipose tissue is often inadequately measured in studies of, for example, breast cancer, and rougher measures such as BMI are used as a proxy, even though this may be inadequate [46]. DXA measurements will make it possible to take these effects into account in a detailed and accurate way. In addition, we collect breast density measures using both automated and subjective methods, enabling comparisons between measurements and future validation of the LIBRA software. The link to NorthPop makes it possible to make use of previously collected data and registry linkages, minimizing costs associated with exposure assessments. Finally, due to the extensive national registers available in Sweden, outcomes in the cohort can be evaluated in depth in the future, and later mammograms can be incorporated continuously.

The main limitation of NorthMom relates to the study size, which is inadequate when it comes to evaluating breast cancer as a primary outcome. However, as studies of environmental effects on breast cancer intermediates such as breast density are scarce, the rare setting of the NorthMom study is likely to enable new discoveries that can be used to infer cancer-specific effects.

## Conclusion

NorthMom is a detailed follow-up of women previously included in the NorthPop Birth Cohort Study [23]. It includes extensive information on environmental exposures during pregnancy and intermediate outcomes related to breast cancer risk, such as breast density. In addition, NorthMom data can be used with high relevance in future studies investigating other outcomes or health-related risk factors.

## Supplementary Information

Supplementary Material 1.

Supplementary Material 2.

## Data Availability

No datasets were generated or analysed during the current study.

## Abbreviations

BMI: Body Mass Index
BI-RADS: Breast Imaging Reporting and Data System
CC: Craniocaudal
DXA: Dual-Energy X-ray absorptiometry
EDCs: Endocrine-disrupting chemicals
GDPR: General Data Protection Regulation
ITS: IT support and system development at Umeå University
KD: Karin Dembrower
LIBRA: Laboratory for Individualized Breast Radiodensity Assessment
LISA: The Longitudinal Integrated Database for Health Insurance and Labor Market Studies
MLO: Mediolateral Oblique
NO_2_: Nitrogen dioxide
NSHDS: Northern Sweden Health and Disease Study
NorthMom: The NorthMom Study
NorthPop: The NorthPop Birth Cohort Study
NKBC: The Swedish Breast Cancer Register
PFAS: Per- and polyfluoroalkyl substances
POPs: Persistent Organic Pollutants
Q-IVF: The National Quality Registry for Assisted Reproduction
VIP: Västerbotten Intervention Programme
WoS: Windows of Susceptibility
