# Supplementary material for: The NorthMom study: population-based follow-up of women in a birth cohort to assess pregnancy exposures and effects on breast cancer risk factors
Source: BMC Public Health. 2026 Sep 24;26:2670. doi: 10.1186/s12889-026-29622-0 (PMC13602515; doi:10.1186/s12889-026-29622-0)
Supplement: Supplementary file 1 [file 12889_2026_29622_MOESM1_ESM.pdf]

SUPPLEMENTARY MATERIAL

The NorthMom study: population-based follow-up of women in a birth cohort to assess pregnancy exposures and effects on breast cancer risk factors.

Alice Fredriksson[1], Richard Lundberg-Ulfsdotter[2], Marie-Therese Vinnars[3], Anna Oudin[4,5], Bethany Van Guelpen[1,6], Magnus Domellöf[2], Christina E. West[2], Wendy Yi-Ying Wu[1], Karin Dembrower[7,8]*, Sophia Harlid[1]*

[1]Department of Diagnostics and Intervention, Oncology, Umeå University, Umeå Sweden
[2]Department of Clinical Sciences, Pediatrics, Umeå University, Umeå, Sweden.
[3]Department of Clinical Sciences, Obstetrics and Gynecology, Umeå University, Umeå Sweden
[4]Department of Epidemiology and Global Health, Umeå University, Umeå, Sweden
[5] Division of Occupational and Environmental Medicine, Department of Laboratory Medicine, Lund University, Lund, Sweden
[6]Wallenberg Centre for Molecular Medicine, Umeå University, Umeå, Sweden.
[7]Breast Imaging Unit, Department of Radiology, Capio Sankt Göran Hospital, Sankt Göransplan, Stockholm, Sweden
[8]Department of Medical Epidemiology and Biostatistics, Karolinska Institutet, Stockholm, Sweden

*Equal contribution

Contents

Invitation letter to NorthMom (translated from Swedish) 2
The Swedish Mammography Screening Program 3
Table S1. NorthMom Questionnaire (translated from Swedish) 4
Figure S1. Mammographic images (examples) 6
References 7

Invitation letter to NorthMom (translated from Swedish)

Invitation to participate in the NorthMom research project

We would like to ask whether you would like to participate in the NorthMom research project. In this document, you will find information about the project and what participation involves.

What is the project, and why were you invited?

The purpose of the NorthMom project is to identify environmental factors that increase the risk of developing breast cancer, primarily before menopause (the time when a woman's menstruation stops). Women who have undergone one or more pregnancies have, in the long term, a lower risk of developing breast cancer compared to women who have never been pregnant, particularly for breast cancer diagnosed after menopause.

However, a completed pregnancy temporarily increases the risk of developing breast cancer before menopause (so-called premenopausal breast cancer). This may be explained by the fact that pregnancy is a period during which breast tissue undergoes major hormonally regulated changes and is therefore especially sensitive to external influences. Pregnancy constitutes a 'window of susceptibility' when environmental exposures are believed to have a greater impact on future cancer risk than if they had occurred at another time.

You are invited to participate in the NorthMom project because you previously took part in the NorthPop study. The data and samples that you provided within NorthPop include information about your living environment and lifestyle during your pregnancy. We wish to reuse these data and samples, as well as collect certain new data from you, to expand our understanding of the mechanisms that drive the development of premenopausal breast cancer. One of the hopes of the project is that the new knowledge generated will help us better identify harmful substances so that they can be reduced in our environment in the future.

The research principal responsible for this research project is Umeå University. The research principal refers to the organization that is responsible for the project. The Swedish Ethical Review Authority has approved the research, and the approval reference number is 2024-04835-01.

Express interest

Scan the QR code below to access a page where you can register your interest in participating in NorthMom. You can also indicate there if you do not wish to be contacted further regarding the project. If you register your interest, you will be asked to provide your contact details so that the Clinical Research Center at Norrland University Hospital can contact you and schedule an appointment. Registering your interest does not entail any obligation to participate.

Participating in NorthMom involves only one visit to the Clinical Research Center, during which you will provide a blood sample, have your weight and height measured, undergo an examination of your body composition (skeleton, muscles, organs, and fat), and complete a short questionnaire. You will also have the opportunity to receive the results of your examination after it has been completed.

No additional visits or questionnaires are included in the project.

The Swedish Mammography Screening Program

In Sweden, mammography screening has been a cornerstone of early detection of breast cancer since the 1980s, and the screening program has successfully contributed to decreasing breast cancer mortality rates by up to 30-40% [1]. The standard-of-care radiological workflow follows the Swedish National Guidelines for mammography screening. Briefly, craniocaudal and mediolateral oblique mammographic views of each breast are acquired using certain mammography equipment. All images are independently assessed by two radiologists, each blinded to the other's assessment. Following Swedish practice, if a suspicious sign is seen, the exam is assessed as abnormal; otherwise, normal. If both readers assess the exam as normal, notification of the negative result is sent. If at least one assessment is abnormal, the case proceeds to a consensus discussion, in which two radiologists review the exam and decide whether to recall for further workup. Recalled women are examined by additional imaging, e.g., special mammography views, tomosynthesis, and ultrasound. If suspicion of cancer remains, a biopsy is acquired; otherwise, the woman is notified of a negative result. Finally, biopsies are analyzed by a pathologist who makes a definitive diagnosis of breast cancer ("screen-detected") or benign tissue. Attendance in the screening program is around 80%, and recall rates are stable at 2-3% [2]. The cancer detection rate for screen-detected cancer is around 4-6/1000 examined women [3].

Table S1. NorthMom Questionnaire (translated from Swedish)

Table S1. NorthMom Questionnaire

Question	Possible responses
Date	DD-MM-YYYY
How many times have you been pregnant (live births, stillbirths, miscarriages, abortions, other)	Dropdown (1-20)
How many pregnancies have you lost before gestational week 12?	Dropdown (1-20)
How many pregnancies have you lost between gestational week 12 and 22?	Dropdown (1-20)
How long did it take you to conceive after starting?	1) Shorter than a year<br>2) Longer than a year<br>3) Don't know, did not actively try to get pregnant
Have you ever sought medical assistance to become pregnant?	1) Yes<br>2) No
How old were you when you started menstruating?	Dropdown (7-20)
Are you still having periods?	1) Yes<br>2) No
When did your last period start?	DD-MM-YYYY
How often do you menstruate?	1) Every month<br>2) Between every 35 days to every 3rd month<br>3) Every 4th to 6th month<br>4) More seldom<br>5) Too irregular to know
If you answered 1 to the question above, what is the approximate length of your cycle?	Dropdown (21-35)
If you no longer menstruate, why is that?	1) Am postmenopausal<br>2) Have removed the uterus<br>3) Due to hormonal birth control, for example an IUD<br>4) Other (text option)
If you are postmenopausal, when was your last period?	Dropdown (20-55)

If you have had a hysterectomy, at what age was that?	Dropdown (20-55)
Have you ever used hormonal contraception?	1) Yes<br>2) No
During which calendar years did you use hormonal contraception?	Multiple slider menus.
Are you currently using hormonal contraception?	1) Yes, combined pills<br>2) Yes, gestagen only pills<br>3) Yes, pills but do not know which kind<br>4) Yes, patches<br>5) Yes, vaginal ring<br>6) Yes, implant<br>7) Yes, injection<br>8) Yes, hormonal IUD<br>9) Yes, other (text option)<br>10) No
If you use birth control pills, do you pause them and get a bleeding?	1) Yes<br>2) Sometimes<br>3) No
How often do you pause your birth control pills to get a bleeding?	1) Every month<br>2) Every other month<br>3) Every third month<br>4) More seldom
Have you ever used hormonal therapy for menopause symptoms?	1) Yes, pills you swallow<br>2) Yes, vaginal suppositories or pills<br>3) Yes, patches<br>4) Yes, spray<br>5) Yes, gel<br>6) Yes, other (text option)<br>7) No
During which calendar years did you use hormonal therapy?	Multiple slider menus.

Figure S1. Mammographic images (examples)

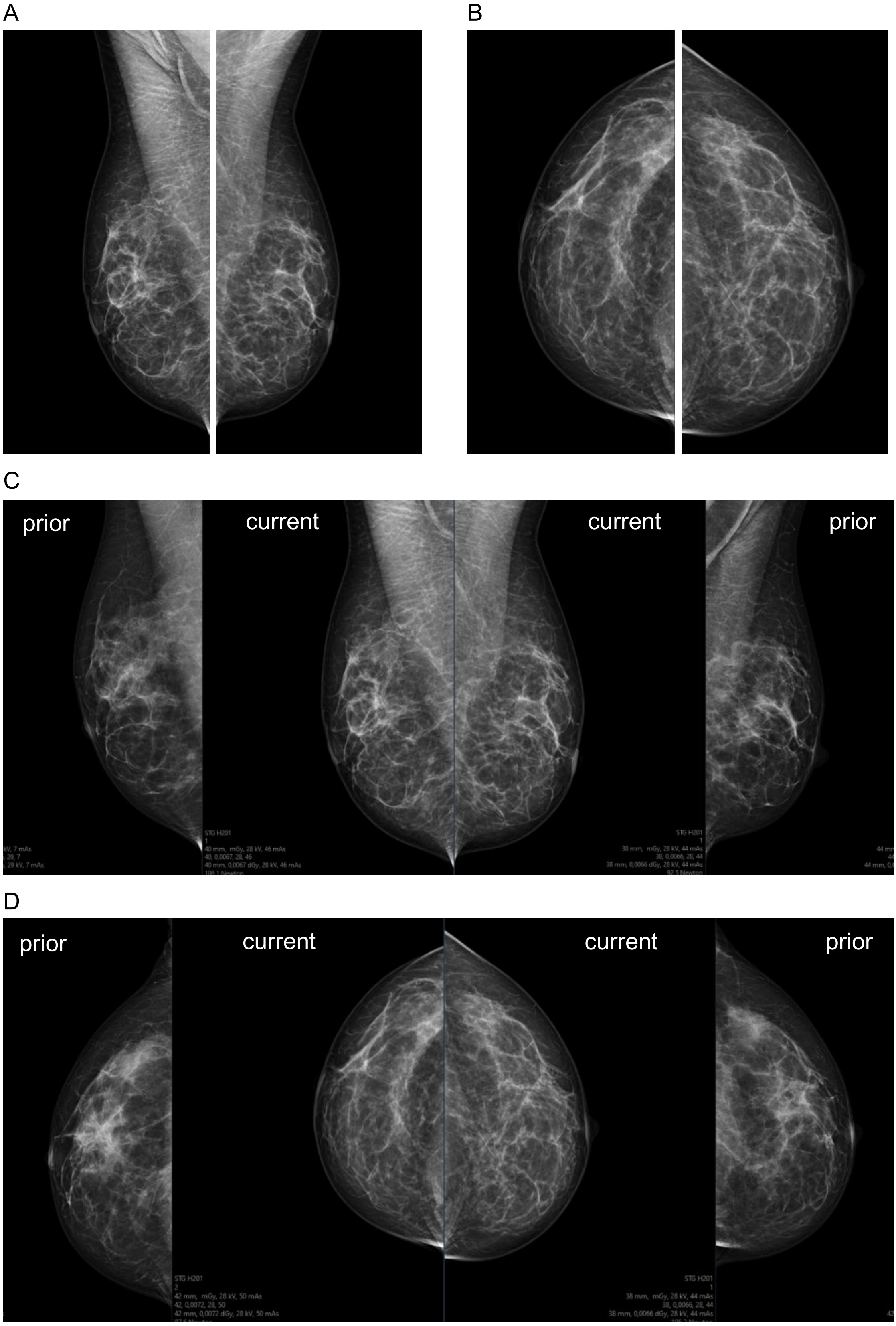

Figure S1. Examples of a normal screening examination (four images, two views).
A) Mediolateral Oblique (MLO) view; B) Craniocaudal (CC) view; C) MLO current and prior examination; D) CC current and prior examination. This example demonstrates a breast classified as category 2 (BI-RADS® 4) or category B (BI-RADS® 5).

References

1. Tabar L, Yen MF, Vitak B, Chen HH, Smith RA, Duffy SW: **Mammography service screening and mortality in breast cancer patients: 20-year follow-up before and after introduction of screening**. *Lancet* 2003, **361**(9367):1405-1410.
2. Lagerlund M, Akesson A, Zackrisson S: **Population-based mammography screening attendance in Sweden 2017-2018: A cross-sectional register study to assess the impact of sociodemographic factors**. *Breast* 2021, **59**:16-26.
3. Zhang Y, Rodriguez J, Mao X, Grassmann F, Tapia J, Eriksson M, Hall P, Czene K: **Incidence and Risk Factors of Interval and Screen-Detected Breast Cancer**. *JAMA Oncol* 2025, **11**(5):519-527.
