# Supplementary material for: The NorthMom study: population-based follow-up of women in a birth cohort to assess pregnancy exposures and effects on breast cancer risk factors
Source: BMC Public Health. 2026 Sep 24;26:2670. doi: 10.1186/s12889-026-29622-0 (PMC13602515; doi:10.1186/s12889-026-29622-0)
Supplement: Supplementary file 2 [file 12889_2026_29622_MOESM2_ESM.pdf]

STROBE Statement—checklist of items that should be included in reports of observational studies

	Item No.	Recommendation	Page No.	Relevant text from manuscript
**Title and abstract**	1	(*a*) Indicate the study's design with a commonly used term in the title or the abstract	1	population-based follow-up
		(*b*) Provide in the abstract an informative and balanced summary of what was done and what was found	2	See methods in abstract
**Introduction**				
Background/rationale	2	Explain the scientific background and rationale for the investigation being reported	4	Breast cancer incidence is increasing among women worldwide, being true for both premenopausal and postmenopausal disease. Global changes to reproductive factors, such as increasing age at first pregnancy and lower parity, may contribute; however, they cannot fully explain the rise in cases.
Objectives	3	State specific objectives, including any prespecified hypotheses	6	… the study aims to improve understanding of the environmental determinants of breast cancer and to inform future prevention and risk stratification strategies
**Methods**				
Study design	4	Present key elements of study design early in the paper	6-7	NorthMom is a new sample and data collection nested within the population-based NorthPop Birth Cohort Study (NorthPop) in Västerbotten County,

				Sweden. ..//.. NorthMom serves as a separate follow-up of the pregnant women recruited to NorthPop, and while the main aim of NorthPop is to investigate exposures during gestation and early life and how they influence children's development, NorthMom examines how exposures during pregnancy affect women's later health.
Setting	5	Describe the setting, locations, and relevant dates, including periods of recruitment, exposure, follow-up, and data collection	7	All women in NorthPop are invited to participate in NorthMom the year after they have turned 40. The list of eligible participants is updated annually to include additional women as they reach the required age.
Participants	6	(*a*) *Cohort study*—Give the eligibility criteria, and the sources and methods of selection of participants. Describe methods of follow-up<br>*Case-control study*—Give the eligibility criteria, and the sources and methods of case ascertainment and control selection. Give the rationale for the choice of cases and controls<br>*Cross-sectional study*—Give the eligibility criteria, and the sources and methods of selection of participants	7, 10	Cohort: All women in NorthPop are invited to participate in NorthMom the year after they have turned 40. Women who have moved out of Västerbotten County, withdrawn from NorthPop, declined further follow-up, or are currently pregnant are considered ineligible.<br>NorthMom may also perform linkages to national registers

				that are separate from those conducted by NorthPop. These include additional linkages to the national prescribed drug register [29], the national cause of death register [30], the Swedish national breast cancer register (NKBC) [31], the national cancer register [32], and the Swedish multigenerational register [33] (in order to obtain data on family history of cancer).
		(*b*) *Cohort study*—For matched studies, give matching criteria and number of exposed and unexposed<br>*Case-control study*—For matched studies, give matching criteria and the number of controls per case		Not relevant
Variables	7	Clearly define all outcomes, exposures, predictors, potential confounders, and effect modifiers. Give diagnostic criteria, if applicable	9, 10,11	Primary outcome: From the Västerbotten health region, NorthMom retrieves radiological images taken as part of the national population-based mammography screening program (further described in the supplementary materials). Screening images are used to classify breast density both automatically and via subjective assessment.<br>Exposures: As all NorthMom women previously participated in NorthPop, extensive

				information about environmental exposures during pregnancy has already been collected prospectively. This data contains information on lifestyle during pregnancy (such as detailed dietary and physical activity data), medications, mental health, and home and work environments, as previously described [24]. Confounders and mediators: Tables 1 and 2
Data sources/ measurement	8*	For each variable of interest, give sources of data and details of methods of assessment (measurement). Describe comparability of assessment methods if there is more than one group	9-11	Tables 1, 2, and 3
Bias	9	Describe any efforts to address potential sources of bias	12	Correlations between BI-RADS assessments, subjective percent density, and automatic density measures are continuously assessed. For women with multiple screening occasions, changes in density measurements over time are also evaluated and documented in NorthMom.
Study size	10	Explain how the study size was arrived at	16	As of the end of 2026, funding for NorthMom will allow recruitment to continue until at least the end of 2028. By then, more than 3 000 women in NorthPop will have turned 40,

	and we should have approximately 1 000 participants in NorthMom (assuming a continued recruitment rate of at least 35 %).

Continued on next page

Quantitative variables	11	Explain how quantitative variables were handled in the analyses. If applicable, describe which groupings were chosen and why	13-14	For analyses of associations between the exposures and continuous breast density (the primary outcome in the study), we conducted a sample size calculation based on breast density at age 40 (performed in R using the ss.SLR() function in the powerMediation package).
Statistical methods	12	(*a*) Describe all statistical methods, including those used to control for confounding	13-14	Data analysis and power calculation section
		(*b*) Describe any methods used to examine subgroups and interactions		Not relevant as no analyses have been performed yet.
		(*c*) Explain how missing data were addressed		Not relevant as no analyses have been performed yet.
		(*d*) *Cohort study*—If applicable, explain how loss to follow-up was addressed<br>*Case-control study*—If applicable, explain how matching of cases and controls was addressed<br>*Cross-sectional study*—If applicable, describe analytical methods taking account of sampling strategy		Not relevant as no analyses have been performed yet.
		(*e*) Describe any sensitivity analyses		Not relevant as no analyses have been performed yet.
**Results**				
Participants	13*	(a) Report numbers of individuals at each stage of study—eg numbers potentially eligible, examined for eligibility, confirmed eligible, included in the study, completing follow-up, and analysed	16	During the first year, NorthMom had a recruitment rate of 36.6% with 442 out of 1207 invited women agreeing to participate (74 women (6.1%) declined to participate, and 691 women (57.2%) did not respond to the invitation or subsequent reminders).
		(b) Give reasons for non-participation at each stage	16	During the first year, NorthMom

				had a recruitment rate of 36.6% with 442 out of 1207 invited women agreeing to participate (74 women (6.1%) declined to participate, and 691 women (57.2%) did not respond to the invitation or subsequent reminders).
		(c) Consider use of a flow diagram	8	Figure 1
Descriptive data	14*	(a) Give characteristics of study participants (eg demographic, clinical, social) and information on exposures and potential confounders		Not relevant as the recruitment is ongoing
		(b) Indicate number of participants with missing data for each variable of interest		Not relevant as the recruitment is ongoing
		(c) *Cohort study*—Summarise follow-up time (eg, average and total amount)		Not relevant as the recruitment is ongoing
Outcome data	15*	*Cohort study*—Report numbers of outcome events or summary measures over time		Not relevant as the recruitment is ongoing
		Case-control study—Report numbers in each exposure category, or summary measures of exposure		Not relevant as the recruitment is ongoing
		Cross-sectional study—Report numbers of outcome events or summary measures		Not relevant as the recruitment is ongoing
Main results	16	(*a*) Give unadjusted estimates and, if applicable, confounder-adjusted estimates and their precision (eg, 95% confidence interval). Make clear which confounders were adjusted for and why they were included		Not relevant as the recruitment is ongoing
		(*b*) Report category boundaries when continuous variables were categorized		Not relevant as the recruitment is ongoing
		(*c*) If relevant, consider translating estimates of relative risk into absolute risk for a meaningful time period		Not relevant as the recruitment is ongoing

Continued on next page

Other analyses	17	Report other analyses done—eg analyses of subgroups and interactions, and sensitivity analyses		Not relevant as the recruitment is ongoing
**Discussion**				
Key results	18	Summarise key results with reference to study objectives		Not relevant as the recruitment is ongoing
Limitations	19	Discuss limitations of the study, taking into account sources of potential bias or imprecision. Discuss both direction and magnitude of any potential bias	17	The main limitation of NorthMom relates to the study size, which is inadequate when it comes to evaluating breast cancer as a primary outcome. However, as studies of environmental effects on breast cancer intermediates such as breast density are scarce, the rare setting of the NorthMom study is likely to enable new discoveries that can be used to infer cancer-specific effects.
Interpretation	20	Give a cautious overall interpretation of results considering objectives, limitations, multiplicity of analyses, results from similar studies, and other relevant evidence		Not relevant as the recruitment is ongoing
Generalisability	21	Discuss the generalisability (external validity) of the study results		Not relevant as the recruitment is ongoing
**Other information**				
Funding	22	Give the source of funding and the role of the funders for the present study and, if applicable, for the original study on which the present article is based	19	NorthMom is supported by grants from the Cancer Research Foundation in Northern Sweden/Lion´s Cancer Research Foundation (grant number: AMP 24-1147), the Swedish Cancer Society (grant numbers: 24 3690 Pj and 26 5140 IA SIA), and the Swedish Research Council (grant

			number: 2024-02868). As part of the funding process, the study underwent external peer review. None of the funders had any specific role in the study conceptualization, design, or data collection.

*Give information separately for cases and controls in case-control studies and, if applicable, for exposed and unexposed groups in cohort and cross-sectional studies.

Note: An Explanation and Elaboration article discusses each checklist item and gives methodological background and published examples of transparent reporting. The STROBE checklist is best used in conjunction with this article (freely available on the Web sites of PLoS Medicine at http://www.plosmedicine.org/, Annals of Internal Medicine at http://www.annals.org/, and Epidemiology at http://www.epidem.com/). Information on the STROBE Initiative is available at www.strobe-statement.org.
